# Response to Aflibercept Therapy in Three Types of Choroidal Neovascular Membrane in Neovascular Age-Related Macular Degeneration: Real-Life Evidence in the Czech Republic

**DOI:** 10.1155/2019/2635689

**Published:** 2019-06-10

**Authors:** Jan Nemcansky, Alexandr Stepanov, Michal Koubek, Miroslav Veith, Yun Min Klimesova, Jan Studnicka

**Affiliations:** ^1^Department of Ophthalmology, Faculty of Medicine, University of Ostrava and University Hospital Ostrava, Ostrava, Czech Republic; ^2^Department of Ophthalmology, Faculty of Medicine in Hradec Kralove, Charles University in Prague and University Hospital Hradec Kralove, Hradec Kralove, Czech Republic; ^3^Department of Ophthalmology, Third Faculty of Medicine, Charles University in Prague and University Hospital Kralovske Vinohrady, Prague, Czech Republic

## Abstract

**Purpose:**

To present a cohort of treatment-naive patients with the neovascular form of age-related macular degeneration (nAMD) treated with aflibercept in a fixed regimen and evaluate the treatment response of three types of choroidal neovascular membrane (CNV)—occult (Type 1), classic (Type 2), and minimally classic (Type 4).

**Methods:**

This was a multicentre, prospective, observational consecutive case series study. Patients diagnosed with three types of CNV of nAMD were treated in a fixed regimen (3 injections every 4 weeks, and then injections at 8 week intervals). The follow-up period was 48 weeks. Best-corrected visual acuity (BCVA) and central retinal thickness (CRT) were measured using Early Treatment Diabetic Retinopathy Study (ETDRS) charts and spectral-domain optical coherence tomography (OCT). The measurements were taken at the baseline and then at 16, 32, and 48 weeks.

**Results:**

The treatment-naive group was composed of 135 eyes of 135 patients in the study. 61 eyes had Type 1 lesions of CNV, 50 eyes had Type 2 lesions, and 24 eyes had Type 4 lesions. Mean baseline BCVA ± SD for Type 1 lesions was 56.1 ± 10.8 ETDRS letters, and then 62.2 ± 12.9 letters, 61.2 ± 13.7 letters, and 62.8 ± 15.1 letters at 16, 32, and 48 weeks, respectively. Mean baseline CRT ± SD for Type 1 lesions was 442.4 ± 194.9 *μ*m, and then 302.5 ± 144.4 *μ*m, 299.7 ± 128.5 *μ*m, and 277.7 ± 106.5 *μ*m at 16, 32, and 48 weeks, respectively. Mean baseline BCVA ± SD for Type 2 lesions was 55.6 ± 9.9 ETDRS letters, and then 62.5 ± 11.1 letters, 60.7 ± 13.0 letters, and 62.5 ± 14.2 letters at 16, 32, and 48 weeks, respectively. Mean baseline CRT ± SD. For Type 4 lesions mean baseline BCVA ± SD was 56.7 ± 9.0 ETDRS letters, and then 59.1 ± 10.6 letters, 59.5 ± 11.4 letters, and 59.2 ± 12.6 letters at 16, 32, and 48 weeks respectively. Mean baseline CRT ± SD for Type 4 lesions was 492.1 ± 187.0 *μ*m, and then 333.3 ± 137.5 *μ*m, 354.4 ± 175.0 *μ*m, and 326.7 ± 122.4 *μ*m at 16, 32, and 48 weeks respectively. All these changes were statistically significant (*p* < 0.005).

**Conclusions:**

The primary outcome of our study is that the treatment with aflibercept in nAMD patients led to statistically significant improvement in BCVA and to a decrease in CRT throughout the follow-up period in both occult and classic types of CNV. The minimally classic type of CNV demonstrated a poorer functional and anatomical response to treatment.

## 1. Introduction

The neovascular form of age-related macular degeneration (nAMD) is a multifactorial chronic degenerative disease affecting the macula and characterized by the presence of a choroidal neovascular membrane (CNV). CNV are classified into four types [1]: Type 1 lesions (occult), when the neovascular membrane is located below the RPE; Type 2 lesions (classic), as it passes through the RPE, compromising the neurosensory retina; Type 3 lesions having been defined as RAP (retinal angiomatous proliferation), which corresponds to neovascularization developed within the neurosensory retina; and Type 4 lesions (minimally classic, Type 1 + Type 2), located below the RPE and above the RPE.

nAMD is well recognized as the main cause of legal blindness in developed countries [2]. Intravitreal antivascular endothelial growth factor (anti-VEGF) injections are currently used as first-line treatment of the disease and are able to stop disease progression and improve visual acuity in most nAMD patients [3–7]. The response to anti-VEGF therapy has been found to be dependent on a variety of factors including the patient's age, baseline best-corrected visual acuity (BCVA), lesion characteristics, and lesion duration [8–11]. There are sufficient data from randomized clinical studies that support the efficacy of aflibercept in both improvement of BCVA and decrease of central retinal thickness (CRT) mainly comparing three types of CNV. However, there is only limited data from real-life clinical settings and protocols, as well as the treatment response in patients from different geographical populations. The aim of this study was to compare the clinical and multimodal imaging features of eyes with Types 1, 2, and 4 CNV lesions at baseline and to assess their comparative visual and anatomical response to intravitreal aflibercept injections in a prospective investigation in real-life clinical settings over a period of 12 months. In fact, to date, there has been no prospective study comparing the effects of intravitreal aflibercept injections on treatment-naive Types 1, 2, and 4 CNV lesions in nAMD.

## 2. Methods

### 2.1. Patient Selection

This was a multicentre, prospective, observational consecutive case series study conducted on patients in regular clinical practice from University Hospital Ostrava, University Hospital Hradec Kralove, and University Hospital Kralovske Vinohrady, Prague, between November 2013 and June 2017. The study has been approved by the institutional review board. Patients at least 50 years of age with treatment-naive, active, nAMD, submacular CNV, less than 12 disk areas with foveal involvement, and evidence of intraretinal fluid and/or subretinal fluid were enrolled for prospective assessment of visual outcomes from baseline to 48 weeks and prospective evaluation of lesion findings using multimodal imaging analysis. Vision criteria for recruitment included Early Treatment of Diabetic Retinopathy Study (ETDRS) BCVA between 50 and 80 letters (Snellen 20/40–20/200) in the affected eye. Further inclusion criteria were aflibercept application in fixed regimen, i.e., beginning of treatment with three monthly doses, followed by four doses bimonthly during the 48-week follow-up. One exception from the dosing regimen in the follow-up period was tolerated, i.e., the minimum number of injections per follow-up period was 6. Exclusion criteria were patients with RAP (Type 3 of CNV), polypoidal choroidal vasculopathy (PCV), bilateral nAMD, and other possible causes of decreased BCVA (e.g., cataract progression and other retinal or anterior segment pathologies). The subtypes of nAMD were defined according to the following official classifications and diagnostic criteria: classification and diagnostic criteria of age-related macular degeneration [12]. Intravitreal injections were administered to all patients under aseptic conditions in the operation room with agreed standards of care used at individual hospital departments.

### 2.2. Data Collection

All subjects underwent BCVA evaluation with the measurement of refraction, anterior segment examination, and indirect ophthalmoscopy of both eyes after pupil dilation with 0.5% tropicamide during the whole of the course of treatment. These examinations were performed at every follow-up visit at 16, 32, and 48 weeks, respectively. BCVA was determined using the standardised ETDRS charts in all three centres according to EMMES protocol. Optical coherence tomography using high-density scans was performed at baseline and then at 16, 32, and 48 weeks. At University Hospital Kralovske Vinohrady, Prague, and at University Hospital Hradec Kralove, CRT was evaluated with an OCT Cirrus 4000 (ZEISS, Oberkochen, Germany), using automatic measurement of central subfield thickness (CST) from macular cube 512 × 128 examination. At University Hospital Ostrava, the examinations were carried out on a Spectralis OCT (Heidelberg Engineering GmbH, Heidelberg, Germany) using automatic analysis of retinal thickness in nine ETDRS subfields, including central subfield (CSF). Images for the analysis were made in 31 horizontal sections, spaced 240 *μ*m apart, with angle 30° × 25°, in HighSpeed mode with noise reduction ART = 9. CSF values measured by Spectralis OCT were then recalculated using the formula published by Krebs et al. [13] so that the examination results from both Cirrus and Spectralis equipment could be assessed together. CRT was defined as the distance between the internal limiting membrane and the presumed RPE at the fovea. All patients signed an informed consent form before the application of intravitreal injections. The study protocol adhered to the tenets of the Declaration of Helsinki principles.

### 2.3. Statistical Analysis

Statistical analysis was performed by means of IBM SPSS Statistics 23 software. The quantitative data are summarized by means of means ± SD, range, and percentage. The BCVA and CRT data were tested by means of the Kolmogorov–Smirnov test on normality, the result of which was the hypothesis of equality was denied. The change of BCVA and CRT measured at baseline and at 16, 32, and 48 weeks was assessed by means of the pairwise Friedman test. The following multiple comparison was performed by means of the pairwise Wilcoxon test adjusted using Bonferroni correction. Statistical significance was defined as *p* < 0.005 (corrected by the Holm–Bonferroni method).

## 3. Results

A total of 135 eyes from 135 treatment-naïve patients were enrolled in the study from 3 centers. Baseline characteristics are presented in Table 1. All patients were treated with aflibercept, nominally 6.5 ± 1.3 injections (minimum 6). 61 eyes had Type 1 lesions (45.2%), 50 eyes had Type 2 lesions (37%), and 24 eyes had Type 4 lesions (17.8%) (*p*=0.5410). The mean age of patients with Type 1 lesions was 73 years (range 59–96) and 41 were women (68%); those with Type 2 lesions had a mean age of 74 years (range 59–88) and 24 were women (49%); and those with Type 4 lesions had mean age of 75 years (range 62–92) and 14 were women (61%) (*p*=0.120). Lesion type had no change after 48 weeks treatment.

### 3.1. Visual Acuity Analysis

**Figure 1:**
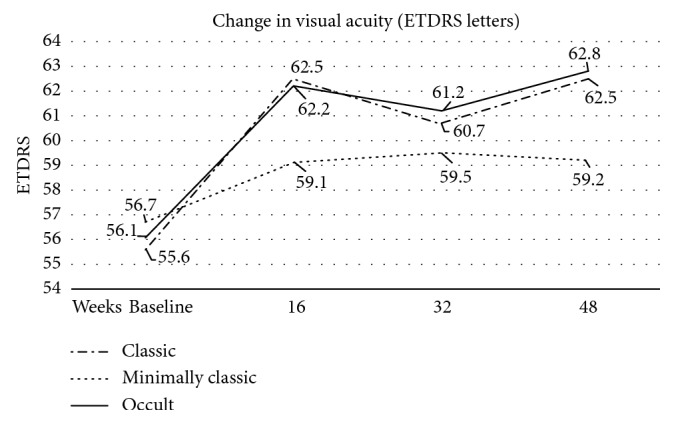
Functional outcomes over time for different types of CNV lesions. All types of CNV lesions showed BCVA improvement from baseline to final 48 week follow-up; however, minimally classic eyes showed a less letter gain in vision.

The baseline ETDRS BCVA ± SD was 56.0 ± 10.2 letters for all lesions, 56.1 ± 10.8 letters for Type 1 lesions, 55.6 ± 9.9 letters for Type 2 lesions, and 56.7 ± 9.0 letters for Type 4 lesions (*p*=0.1780). BCVA values and change are shown in Figure 1. The BCVA at final 12-month follow-up was 62.0 ± 14.45 letters for all lesions, 62.8 ± 15.1 letters for Type 1 lesions, 62.5 ± 14.2 letters for Type 2 lesions, and 59.2 ± 12.6 letters for Type 4 lesions, respectively (*p*=0.1350). Changes compared to baseline values were statistically significant at 16, 32, and 48 weeks (*p* < 0.005). The most significant change was recorded at 48 weeks in Type 1 lesions of CNV (+6.7 letters from baseline) in Table 2. In Type 2 lesions of CNV, the most significant change was recorded after the first three aflibercept injections at the 16^th^ week follow-up and at the end of one-year treatment (+6.9 letters from baseline). The most significant change was recorded at the 32^th^ week in Type 4 lesions of CNV (+2.8 letters from baseline). The ratio of patients with improvement in BCVA (over 15 letters) was 27.9% for Type 1 lesions, 30.0% for Type 2, and 4.2% for Type 4, and these changes were significant in all cases (*p* < 0.005). The ratio of patients with deterioration in BCVA (over 15 letters) was 3.3% for Type 1 lesions, 2.0% for Type 2, and neither patient for Type 4 (not significant changes).

### 3.2. Anatomic Outcomes

**Figure 2:**
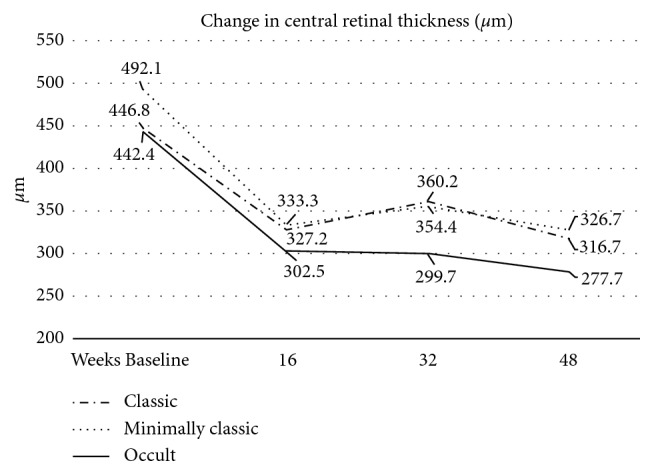
Anatomical outcomes over time for different types of CNV lesions. All types of CNV lesions showed a significant decrease of CRT from baseline to final 48 week follow-up. The most significant decrease is after the initial 3 aflibercept injections.

Baseline CRT ± SD was 452.9 ± 182.60 *μ*m for all lesions, 442.4 ± 194.9 *μ*m for Type 1 lesions, 446.8 ± 159.1 *μ*m for Type 2 lesions, and 492.1 ± 187.0 *μ*m for Type 4 lesions (*p*=0.2160). CRT values and change are shown in Figure 2. CRT ± SD at the final 12-month follow-up was 301.0 ± 124.73 *μ*m for all lesions, 277.7 ± 106.5 *μ*m for Type 1 lesions, 316.7 ± 139.1 *μ*m for Type 2 lesions, and 326.7 ± 122.4 *μ*m for Type 4 lesions (*p*=0.1850). The most significant change was recorded at the end of one year of treatment in all types of CNV. For Type 1 lesions, it was −164.7 *μ*m from baseline, for Type 2 lesions −130.1 *μ*m, and for Type 4 lesions −165.4 *μ*m from baseline, respectively (Table 2). The decrease in CRT in all groups was statistically significant (*p* < 0.005). At the end of follow-up, residual macular fluid was present in 27.8% of all patients, of whom 23.8% had Type 1 lesions, 32.7% had Type 2 lesions, and 43.5% had Type 4 lesions.

Additional treatment criteria included the signs of exudation (persistent or new subretinal or intraretinal fluid on SD-OCT), and the number of patients with additional treatment was 53 (39.3%).

Throughout the follow-up period, no significant ocular or systemic side effects of the treatment were recorded.

## 4. Discussion

In this retrospective study, the baseline functional and anatomic characteristics of treatment-naive eyes with neovascular AMD with Types 1, 2, or 4 CNV during a 1-year response to intravitreal aflibercept injections were evaluated in routine clinical practice in the Czech Republic. A relatively large cohort (*n* = 135) of treatment-naïve patients was enrolled, and a BCVA gain of 6.4 letters was achieved for the whole group of patients after a regular treatment regimen.

Jung et al. in his study noted that 40% of all lesions were Type 1, whereas 34% were Type 3 and 17% were Type 4 lesions [14]. The remaining lesions were Type 2 (9%). In a separate study, Marsiglia et al. found that 47.0% of unilateral treatment-naive eyes with neovascular AMD harbored Type 1 lesions, 24.1% harbored Type 3 lesions, 12.0% had Type 2, and 16.9% had Type 4 lesions [15]. According to the PERSEUS study (The Prospective Noninterventional Study to Assess the Effectiveness of Aflibercept in Routine Clinical Practice in Patients with Neovascular AMD), 25.4% patients had Type 1 lesions, 24.9% patients had Type 2 lesions, and 4.6% patients had Type 4 lesions [16]. Our study found a distribution of 44.4% with Type 1 lesions, 36.3% with Type 2 lesions, and 17.3% with Type 4 lesions. The predominance of Type 1 CNV in our study correlates with the works of Marsiglia, Jung, and the PERSEUS study. The higher proportion of Type 2 lesions in our study may be allied to our moderate sample size.

In terms of BCVA, at the end of one year's treatment, our patients had a gain of 6.7 letters in Type 1 lesions, a gain of 6.9 letters in Type 2 lesions, and a 2.8-letter gain in Type 4 lesions. The studies of Marsiglia and Jung have shown similar visual acuity improvements [14, 15]. All groups demonstrated statistically significant improvements in vision, but eyes with Type 4 lesions demonstrated a worse letter gain than eyes with Types 1 and 2 lesions. The minimally classic form of CNV (Type 4) is less treatable for severe morphological changes of the retina, which corresponds to our results.

We establish that, in our study, 93.1% of all groups of patients maintained visual acuity (loss of less than 10 ETDRS letters from baseline) at the end of one-year treatment, which is proportionate to the results of the VIEW study with one-year aflibercept treatment in a fixed regimen [5]. In our study, the mean gain was 6.0 letters at one-year follow-up when patients received 6.5 ± 1.3 mean injections. This correlates with 8.4 letters in the analysis of the VIEW clinic study when patients received 7.5 ± 1.2 mean injections at 48 weeks [5]. The ratio of patients with improvement in BCVA over 15 letters was 30.6% for the whole group of patients [16]. Unfortunately, no comparison was described in each CNV classification. Our results for Types 1 and 2 CNV lesions are comparable with the VIEW study.

In the 1-year outcomes of the PERSEUS study, the regularly treated patients (2 mg intravitreal aflibercept every 2 months) gained an average of 8.0 letters with 7.4 mean injections during the first year with the ratio 32.0%, whereas the whole group of treatment-naïve patients experienced a gain of 15 letters or more compared with baseline [16]. Unfortunately, no comparison was described in each CNV classification. We explain the smaller gain of ETDRS letters in our study by fewer groups of patients and a smaller number of intravitreal injections of aflibercept.

The results from our study are also in line with Eleftheriadou et al. [17], who published a meta-analysis of real-world outcomes of aflibercept for nAMD including 102 patients. The mean change in BCVA for patients receiving a fixed regimen for the first year of aflibercept treatment was 5.4 ETDRS letters, when patients received 7.3 ± 1.7 mean injections in year 1. The results from our study also correlate with those from Talks et al. [18], who published the first year outcomes of a fixed treatment regimen using aflibercept for nAMD. Talks and associates reported a mean gain of 5.1 ETDRS letters in comparison with the gain of 6.0 letters in our study, with a mean number of 7.5 ± 1.4 injections, whereas in our study, the reported mean numbers of injections were 6.5 ± 1.3 injections in the first year.

The ratio of patients with statistically significant improvement in BCVA (over 15 letters) in our study was 27.9% for Type 1 lesions, 30.0% for Type 2, and 4.2% for Type 4. Based on results of BCVA changes, we can conclude that eyes with Type 4 lesions demonstrated a worse letter gain than eyes with Type 1 and Type 2 lesions, so the CNV type can be a predictor to the change of BCVA after anti-VEGF treatment. The minimally classic form of CNV (Type 4) demonstrated a poorer functional and anatomical response to anti-VEGF treatment due to severe morphological changes of the retina.

Morphologically, our patients demonstrated a significant reduction of baseline CRT at the final 12-month follow-up in all types of CNV. Types 1 and 4 lesions resolved more rapidly with anti-VEGF therapy during the treatment, resulting in a smaller difference in thickness between these 2 lesion types at final follow-up. Reduction of CRT in the case of Type 2 lesions displayed a much smaller decrease than the other types of lesions. Such a result can be explained by more significant baseline morphological changes and intraretinal fluid associated with Type 2 lesions. Residual macular fluid was present in 43.5% of Type 4 lesions at the final 12-month follow-up, which statistically corresponds with the poorer improvement in BCVA for this type of CNV lesion (*p* < 0.005).

In the study of Chen et al., the mean baseline CRT for Type 1 lesions was 226 ± 99 *μ*m with a consequent reduction of 13 ± 46 *μ*m after 1 year of aflibercept treatment [19]. Analysis of retinal morphology on SD-OCT showed that residual macular intraretinal and/or subretinal fluid was present in 93.0% of eyes with Type 1 lesions at baseline and 31.0% at year 1 [19].

In our study, the mean baseline CRT for Type 1 lesions was 442.4 ± 194.9 *μ*m and mean CRT reduction was −164.7 ± 57.9 *μ*m at the end of the first year of follow-up. Residual macular fluid was present in 30.0% of Type 1 lesions at the end of follow-up. We attribute the greater decrease in CRT in our study to the higher baseline CRT.

The mean reduction of CRT for all types of CNV lesions in our study was 151.9 *μ*m at 12 months, compared with a mean reduction of 139 *μ*m in the integrated analysis of the VIEW study at 52 weeks [5]. In our study, retinal fluid was not presented in 72.2% of eyes at year 1. According to the VIEW study, 67.7% of eyes were free of macular fluid at year 1. Our data are similar to those of the VIEW clinical trial in relation to morphologic changes. In our study, we had a higher proportion of eyes without macular fluid after 1 year of aflibercept treatment in real-life evidence in comparison with VIEW, and this can be explained as a chance finding in our group of patients. Moreover, this difference may in part be supposed for by the more precise standards used in a reading center when ruling out the presence of retinal edema.

The advantages of our study have an effort to reduce inclinations by recording and collecting SD-OCT results relating to retinal morphology and CRT. This procedure differs with retrospective reports from other real-life studios [18, 20], which aim attention at BCVA without publishing any data from OCT examination. Another strength point of our study is the multicenter design. Patients were enrolled in three university municipal hospitals throughout the Czech Republic. The limitations of our study are the retrospective and observational nature and the relatively small sample size compared with bigger clinical trials.

## 5. Conclusion

We present one-year real-life outcomes in treatment-naive patients with three types of CNV lesions in the neovascular form of AMD treated with aflibercept in a fixed regimen. The primary outcome of our study is that aflibercept treatment with a fixed regimen results in great visual acuity gain and improvement in retinal anatomic outcomes over a one-year follow-up in occult and classic types of CNV. The minimally classic type of CNV demonstrated a poorer functional and anatomical response to treatment. So, the CNV type can be a predictor to the change of BCVA after anti-VEGF treatment.

## Figures and Tables

**Table 1:** Baseline and anatomic demographics and vision of all included eyes separated by CNV type.

	All patients (*n* = 135) (100%)	Type 1 (*n* = 61) (44.4%)	Type 2 (*n* = 50) (36.3%)	Type 4 (*n* = 24) (19.3%)	*p*
Mean age, yrs	74.2 ± 7.3	73.7 ± 7.6	74.2 ± 6.6	75.3 ± 7.3	0.120
Female, no.	79 (59%)	41 (68%)	24 (49%)	14 (61%)	0.672
CRT (*μ*m)	452.9 ± 181.9	442.4 ± 194.9	446.8 ± 159.1	492.1 ± 187.0	0.216
BCVA (ETDRS)	56.0 ± 10.2	56.1 ± 10.8	55.6 ± 9.9	56.7 ± 9.0	0.178

**Table 2:** Mean change in ETDRS letters and CRT in micrometers from baseline for different types of CNV lesions during the first year of intravitreal aflibercept injection treatment.

Types of CNV	ETDRS letters			CRT (micrometers)		
Weeks	16	32	48	16	32	48
Classic	6.9	5.1	6.9	119.6	86.6	130.1
Minimally classic	2.4	2.8	2.5	158.8	137.7	165.4
Occult	6.1	5.1	6.7	139.9	142.7	164.7

Classic CNV eyes showed the greatest letter gain in vision yet after the initial 3 aflibercept injections. Minimally classic CNV eyes showed a great decrease of CRT.

## Data Availability

The data used to support the findings of this study are freely available and are included within the article.

## Additional Points

*Summary.* Patients with the neovascular form of age-related macular degeneration were treated with aflibercept in a fixed regimen. After 12-month follow-up, significant improvement in both morphological and functional results was observed in the three types of choroidal neovascular membrane.
